# Antitumor Activity of *Ficus deltoidea* Extract on Oral Cancer: An In Vivo Study

**DOI:** 10.1155/2020/5490468

**Published:** 2020-02-10

**Authors:** May Al-koshab, Aied M. Alabsi, Marina Mohd Bakri, Rola Ali-Saeed, Manimalar Selvi Naicker

**Affiliations:** ^1^Department of Oral & Craniofacial Sciences, Faculty of Dentistry, University of Malaya, Kuala Lumpur 50603, Malaysia; ^2^Department of Oral Biology and Biomedical Sciences, Faculty of Dentistry, MAHSA University, Jenjarom 42610, Selangor, Malaysia; ^3^Department Pathology, Faculty of Medicine, University of Malaya, Kuala Lumpur 50603, Malaysia

## Abstract

**Background:**

The aim of this study is to evaluate the chemopreventive and chemotherapeutic activities of *Ficus deltoidea* (FD) in an animal model induced for oral cancer using 4-nitroquinoline-1-oxide (4NQO).

**Methods:**

Male Sprague-Dawley (SD) rats were randomized into six groups (*n* = 7 per group): Group 1 (untreated group); Group 2 (control cancer group) received 4NQO only for 8 weeks in their drinking water; Groups 3 and 4 (chemopreventive) received 4NQO for 8 weeks and were simultaneously treated with FD extract at 250 and 500 mg/kg, respectively, by oral gavage; Groups 5 and 6 (chemotherapeutic) received 4NQO for 8 weeks followed by the administration of FD extract at 250 and 500 mg/kg, respectively, for another 10 weeks. The incidence of oral cancer was microscopically evaluated. Moreover, immunohistochemical expression was analysed in tongue specimens using an image analyser computer system, while the RT^2^ profiler PCR array method was employed for gene expression analysis.

**Results:**

The results of the present study showed a beneficial regression effect of the FD extract on tumor progression. The FD extract significantly reduced the incidence of oral squamous cell carcinoma (OSCC) from 100% to 14.3% in the high-dose groups. The immunohistochemical analysis showed that the FD extract had significantly decreased the expression of the key tumor marker cyclin D1 and had significantly increased the expression of the *β*-catenin and e-cadherin antibodies that are associated with enhanced cellular adhesion. Based on the gene expression analysis, FD extract had reduced the expression of the *TWIST1* and *RAC1* genes associated with epithelial-mesenchymal transition (EMT) and had significantly downregulated the *COX-2* and *EGFR* genes associated with cancer angiogenesis, metastasis, and chemoresistance. Our data suggest that the FD extract exerts chemopreventive and chemotherapeutic activities in an animal model induced for oral cancer using 4NQO, thus having the potential to be developed as chemopreventive and chemotherapeutic agents.*β*-catenin and e-cadherin antibodies that are associated with enhanced cellular adhesion. Based on the gene expression analysis, FD extract had reduced the expression of the *TWIST1* and *RAC1* genes associated with epithelial-mesenchymal transition (EMT) and had significantly downregulated the *COX-2* and *EGFR* genes associated with cancer angiogenesis, metastasis, and chemoresistance. Our data suggest that the FD extract exerts chemopreventive and chemotherapeutic activities in an animal model induced for oral cancer using 4NQO, thus having the potential to be developed as chemopreventive and chemotherapeutic agents.*TWIST1* and *RAC1* genes associated with epithelial-mesenchymal transition (EMT) and had significantly downregulated the *COX-2* and *EGFR* genes associated with cancer angiogenesis, metastasis, and chemoresistance. Our data suggest that the FD extract exerts chemopreventive and chemotherapeutic activities in an animal model induced for oral cancer using 4NQO, thus having the potential to be developed as chemopreventive and chemotherapeutic agents.*RAC1* genes associated with epithelial-mesenchymal transition (EMT) and had significantly downregulated the *COX-2* and *EGFR* genes associated with cancer angiogenesis, metastasis, and chemoresistance. Our data suggest that the FD extract exerts chemopreventive and chemotherapeutic activities in an animal model induced for oral cancer using 4NQO, thus having the potential to be developed as chemopreventive and chemotherapeutic agents.*COX-2* and *EGFR* genes associated with cancer angiogenesis, metastasis, and chemoresistance. Our data suggest that the FD extract exerts chemopreventive and chemotherapeutic activities in an animal model induced for oral cancer using 4NQO, thus having the potential to be developed as chemopreventive and chemotherapeutic agents.*EGFR* genes associated with cancer angiogenesis, metastasis, and chemoresistance. Our data suggest that the FD extract exerts chemopreventive and chemotherapeutic activities in an animal model induced for oral cancer using 4NQO, thus having the potential to be developed as chemopreventive and chemotherapeutic agents.

## 1. Introduction

The third most common cancer in Malaysian Indian community is oral cancer [1], and it is highly associated with the practice of betel quid chewing, tobacco smoking, and excessive alcohol consumption [2]. Microorganisms that are present in the oral cavity can metabolize alcohol by enzymatic activity to acetaldehyde which is a known carcinogen [3]. Another well-known and potent carcinogen present in cigarette smoke [4], 4NQO, has been shown to induce oral cancer in animals when consumed in drinking water. The animal model for induction of oral cancer by 4NQO is widely used by the researchers for studying the process of carcinogenesis and for evaluating the effects of natural products on cancer development [5–7]. Over the years, the use of chemotherapeutic drugs such as cisplatin or allopathy medicine for treating cancer has been successful. However, this type of treatment modality is often associated with chemotherapeutic drugs toxicity, resulting in severe side effects. Moreover, around 90% of drug failures in metastatic cancers are caused by chemoresistance [8]. As a result, researchers have tried to look for other types of treatment modalities and these include the use of natural products for treating cancer.

*Ficus deltoidea* (FD) is one of the common medicinal plants used in Malaysia and other Southeast Asia countries. In Malaysia, the FD extract is used traditionally to heal wounds, sores, and rheumatism and can also be used as an antidiabetic drug or as an after-birth tonic. Despite all these traditional claims, scientific studies of this plant are very limited with most studies focusing on evaluating its antioxidant, antihyperglycemic, antinociceptive, antihypertensive, wound, and ulcer-healing properties [9]. It was reported in a study that 85 percent of the overall antioxidant activity of the FD extract was attributable to the flavan-3-ol monomers and the proanthocyanidins [10].

The anticancer activity of the FD extract against the human ovarian carcinoma cell line using a cell-based assay has been demonstrated [11]. Ware et al. [12] reported that the FD extract possesses potent natural antioxidant and anticancer activity when tested on prostate cancer DU145 cell line. Norrizah et al. [13] observed that the cytotoxic activity against the HL-60 cell line of the FD Leaf extract was more potent than the fruit extract. Besides that, when tested against the male reproductive system of the rats, there was a significant effect of the FD extract on the testes and epididymis weight, sperm count, and sperm viability [13]. There have been no in vivo studies showing the effects of FD extract towards oral cancer. Hence, this study was conducted to assess the chemopreventive and chemotherapeutic activities of the FD aqueous extract in an animal model induced for oral cancer using 4NQO.

## 2. Materials and Methods

The study was approved by the Institutional Animal Care and Use Committee (IACUC), University Malaya (Ethic reference no. 2016-190607/DENT/R/AMHS).

All experimental procedures were performed according to the FOM IACUC guidelines. All rats received human care based on the criteria summarized in the “*Guide for the care and use of laboratory animals*” (USA) [14].

### 2.1. 4-Nitroquinoline-1-oxide and Aqueous Extract of *Ficus deltoidea*

4-Nitroquinoline-1-oxide (4NQO) is a carcinogenic chemical. 4NQO may naturally occur in the environment but is typically manufactured for research purposes. 4NQO (Cas No. N-8141, Sigma Aldrich) was purchased from Labchem Sdn. Bhd., Malaysia.

The aqueous extract of FD was purchased from HCA Products Sdn Bhd. University Putra Malaysia, Selangor, Malaysia, with voucher number U1578/15. The aqueous extraction was done using FD leaves obtained from Herbagus Sdn Bhd according to the standardized 100% pure extraction method. The leaves of the plant were dried and powdered mechanically. The dried powdered leaves (100 g) were infused in distilled water at 60°C for 4 hours and filtered, and the liquid extract content was spray-dried for 6 hours at 80°C.

### 2.2. Animals

A total of 42 healthy male Sprague-Dawley (SD) rats (6–8 weeks old with body weight between 200–250 g) were obtained from the Animal Experimental Unit, Faculty of Medicine, University of Malaya, Kuala Lumpur. The animals were maintained under environmental condition of the animal house and had free access to standard ad libitum rat chow diet and fresh reverse osmosis (RO) water. They were housed in animal cages in an air-conditioned area at 22 ± 30°C and with a 12 h light/dark cycle in an experimental room. In order to ensure the animals were adapted to the laboratory condition, they were housed at the animal unit for 7 days before the experiments.

### 2.3. Dose Selection

The dose of 4NQO used in the present study was 20 ppm, and this dose was chosen based on many previous studies as it has been shown that oral cancer was successfully induced in Sprague Dawley rat model when 20 ppm of 4NQO agent dissolved in drinking water was administrated for 8 weeks [15, 16].

In this study, the doses of 250 and 500 mg/kg were chosen for the FD extract based on the previous studies. The dose of 2500 mg/kg when used in a subchronic toxicity study was discovered to be nontoxic [17, 18]. Thus, we have chosen to use 1/10th of maximum tolerable dose for therapeutic purpose [19], which were 250 and 500 mg/kg representing the low and higher doses.

### 2.4. Study Design

#### 2.4.1. Induction of Oral Carcinogenesis by 4NQO

4-Nitroquinoline-1-oxide (4NQO), a water-soluble quinoline derivative, can produce tongue neoplastic and preneoplastic lesions. Drinking water containing 4NQO was freshly prepared twice a week in RO water and was administered to the rats in light-shielded water bottles (to protect the prepared solution from any unwanted effect of light exposure) at the concentration of 20 ppm for 8 weeks [16].

#### 2.4.2. Chemopreventive Study

In order to evaluate the effect of the FD extract during the initiation phase of 4NQO-induced oral carcinogenesis, a chemopreventive study was designed in male SD rats. The rats were randomized into 4 groups of 7 rats per group. Group 1 was given normal RO water (untreated control), while the remaining 3 groups of rats (Groups 2, 3, and 4) were given 4NQO (Sigma Aldrich) solution as drinking water for 8 weeks as previously described [20, 23]. The 3 groups also received vehicle (water), FD extract at 250 and 500 mg/kg, respectively, daily in a volume of 10 ml/kg body weight, starting one week before the consumption of 4NQO, and this was continued for another week when the consumption of 4NQO has completed. Following this, the animals in all groups were switched back to RO water, and this continued until the end of this study at 22 weeks.

#### 2.4.3. Chemotherapeutic Study

**Figure 1:**
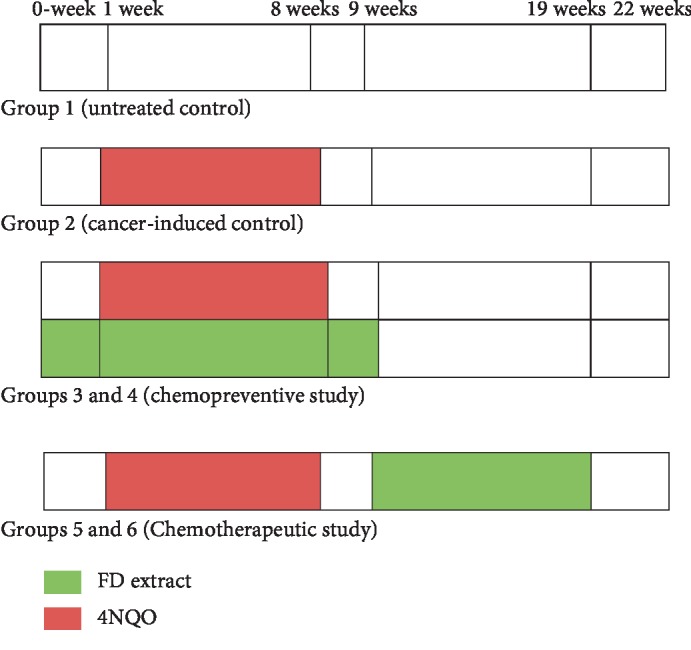
Diagrammatic representation of experimental protocol for administration of 4NQO and FD extract in male Sprague-Dawley rats.

To evaluate the effect of the FD extract in the postinitiation phase of the oral cancer induction using 4NQO in an animal model, chemotherapeutic study was designed. A total of 14 male SD rats were randomized into 2 groups (Group 5 and 6) of 7 rats per group. The rats from groups 5 and 6 were given 4NQO (Sigma Aldrich) solution as drinking water for 8 weeks. Following this at the 9th week, Groups 5 and 6 were administrated with the FD extract at 250 and 500 mg/kg respectively, for another 10 weeks. The FD administration started 1 week after the cessation of the 4NQO treatment [20, 21]. Both of the control groups (Group 1—untreated and Group 2—cancer induced) that were involved in the chemopreventive study were also used in the chemotherapeutic study since both experiments were carried out at the same time (Figure 1). For both chemopreventive and chemotherapeutic studies, the observation of the overall health and behavior of the rats was done once a day to identify possible toxicities that may have resulted from the administration of the 4NQO carcinogen. The body weight of the rats was recorded every week during the study. The experiment was terminated on the 22nd week. General anesthesia was induced through the administration of an intramuscular injection of 5 mg/kg of xylazine 100 mg/ml and 50 mg/kg ketamine 100 mg/ml. As previously described by Stokes et al. [22], the rats were sacrificed through cervical dislocation and was followed by the excision of the whole tongue.

### 2.5. Tumor Volume and Histopathological and Immunohistochemical Analysis

The volume of the animal tongues tumor was recorded after the experimental period and were measured based on the formula (length × width × height × *π*/6) as mentioned in a previous study [23]. The tongue sections were analysed histologically and classified as squamous cell carcinoma, dysplasia, hyperplasia, or normal, per animal [5]. Immunohistochemical evaluation was conducted in the present study to determine the effect of the FD extract, the expression of tumor markers, cyclin D1, bcl2, p53, *β*-catenin, and e-cadherin in the tongue tissue sections of the Sprague-Dawley rats. Formalin-fixed, paraffin-embedded tissue sections (4 *μ*m) were cut from the paraffin blocks and deparaffinized and rehydrated. The antibodies were then applied according to the manufacturer's protocol at the pathology laboratory, Pathology Department, University of Malaya. Immunohistochemistry was conducted using the Ventana Benchmark XT auto stainer with the following antibodies: p53 (clone DO-7, Dako Japan), e-cadherin (clone NCH-38, Dako Japan), *β*-catenin (clone b-Catenin-1, Dako Japan), bcl2 (clone 124, Dako Japan), and cyclin D1 (clone SP4, Thermo Fisher Scientific). The IHC staining was carried out based on the automated process for routine staining at the pathology laboratory. The IHC computed analysis using Image J software was as explained in our previous work [24].

### 2.6. RT^2^ PCR Array

Purification of total RNA extracted from the tongue specimens was carried out using RNeasy Protect Mini kit (Qiagen, USA). Thirty milligram of tongue specimens kept at −80°C and thawed was homogenized, and the RNA was isolated using the QIAzol Lysis reagent based on the manufacturer's instructions. Measurements of the concentration and purity of RNA were obtained by using a NanoDrop (BioTek, USA). The RNA was then kept at −80°C. Reverse transcription (RT) of RNA to single-stranded cDNA was executed using the RT^2^ first strand kit (Qiagen), according to the manufacturer's guidelines. The cDNA was then mixed with RT^2^ SYBR Green/ROX qPCR Master mix (Qiagen, USA), and the mixture was added into a 96-well RT^2^ PCR Array according to manufacturer's instruction. Cycle threshold (Ct) values were used for calculations of fold changes using the 2−∆∆Ct method.

### 2.7. Reliability of Measurements

The Reliability of the histological results has been done by SPSS software using the Kappa test. The pathologist has reassessed each slide blindly twice, with one-week interval. The results showed an agreement among the readings, *K* = 0.476, *p* < 0.001.

### 2.8. Statistical Analysis

The data were recorded and analysed using the SPSS version 20. All assays were conducted in at least five separate experiments. The quantitative results were expressed as the mean ± SD. Data representing the IHC analysis, body weight, and tumor size were compared using the one-way ANOVA test with post hoc comparisons made using the Dunnetts test. The histological qualitative data were analysed using the chi-square statistical test. The data obtained using the RT^2^ profiler PCR array were analysed using the Gene Globe Data Analysis Center on QIAGEN's website (http://www.qiagen.com/my/shop/genes-and-pathways/data-analysis-center-overview-page/). The results were statistically significant at *p* value ˂0.05.

## 3. Results

### 3.1. Body Weight

The mean body weight was significantly increased in the FD 500 mg/kg group (group 4) and normal group (group 1) when compared with the 4NQO group (group 2), (*p*=0.09 and *p* < 0.001 using the post hoc test). No significant differences were shown between the 4NQO control group and other groups (Table 1).

### 3.2. Tongue Tumor Volume

FD-treated groups (group 3 and 4) showed a significant lower size of developed tumor compared with group 2 induced by 4NQO (Table 2). The post hoc test showed *p* values of 0.023 and <0.001, respectively.

### 3.3. Histological Analysis

Histological evaluations were performed blindly with light microscopy by a qualified pathologist. The tongue tissue sections were assessed and classified as squamous cell carcinoma, dysplasia, hyperplasia, or normal per animal. Normal oral mucosa of the tongue squamous epithelium was observed in tongue tissues of the normal group animals. Hyperplasia and dysplasia have been detected in the animals subjected to 4NQO. In the control cancer group, the incidence of OSCC was 85.7%. Following treatment with the FD extract at 250 and 500 mg/kg, for the chemopreventive study, the OSCC incidence was reduced significantly (*p* < 0.05) at 42.9% and 14.3%, respectively.

The diagnosis of each tongue sample was based on the final histopathological changes. For each rat specimen that was diagnosed as having OSCC, the presence of hyperplasia and dysplasia lesions could still be detected in some parts of the same tongue.

The dysplastic lesions that developed from the chemopreventive groups were observed to be 14.6% in the 4NQO cancer group, while the FD extract groups (treated with 250 and 500 mg/kg) were 42.9% and 28.6%, respectively. Hyperplasia incidence in the FD extract groups treated at 250 and 500 mg/kg were 14.3% and 57.1%, respectively.

In the chemotherapeutic groups, the incidence of OSCC in rats treated with FD extract at 250 and 500 mg/kg was 28.6% and 14.3%, respectively. The incidence of dysplasia lesions was 14.3% in the control cancer group of 4NQO, while that in the FD extract group treated with 250 and 500 mg/kg was 71.4% and 85.7%, respectively. Hyperplasia lesions in the FD and 4NQO groups were 0%. Generally, it is observed that the administration of FD extract especially at the highest dose (500 mg/kg) had reduced the incidence of hyperplasia, dysplasia, and squamous cell carcinoma significantly in both chemopreventive study and chemotherapeutic study (*p*=0.03) (Table 3).

### 3.4. Immunohistochemical Analysis

**Figure 2:**
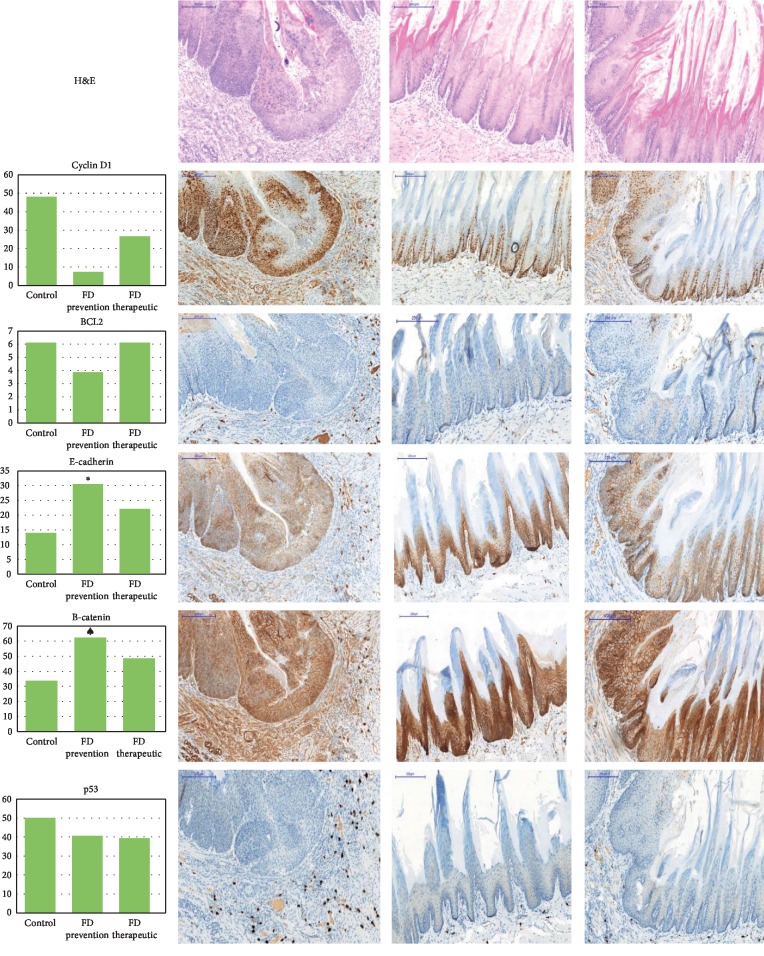
Bar chart and photomicrographs of animals stained with H & E staining and immunohistochemistry stains (cyclin D1, bcl2, e-cadherin, *β*-catenin, and p53, respectively) of both treated groups and control group.

Immunohistochemical evaluation of the selected IHC stains was conducted to assess and compare positive stained percentage area between the control group and the treated groups after the end of 4NQO administration (Figure 2). Based on the parametric one-way ANOVA, there was a significant difference in the mean percentage of cyclin D1, *β*-catenin, and e-cadherin in treated groups compared with the untreated group. However, bcl2 and p53 showed nonsignificant expression in treated groups when compared with the control cancer group. A post hoc Tukey test was used to detect the differences between the groups as presented in Table 4.

### 3.5. RT^2^ PCR

**Figure 3:**
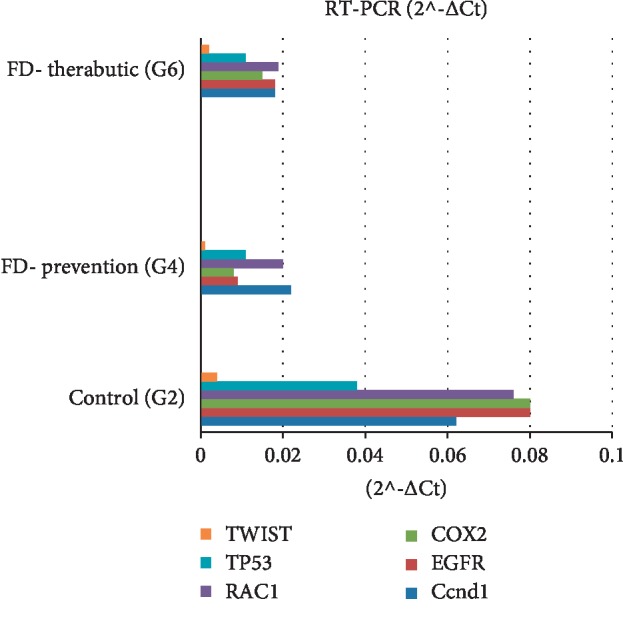
Expression of selected genes in control (G2) and FD-treated groups (G4 and G6) by RT^2^ PCR. The bars indicate the 2^−ΔCt value of *TWIST1*, *TP53*, *RAC1*, *COX-2*, *EGFR*, and *CCND1* genes in the G2, G4, and G6 groups.

A custom RT^2^ PCR array was carried out to evaluate the alteration of gene expression between the control cancer group of animals (group 2) and FD-treated groups in both chemoprevention and chemotherapeutic studies. Gene expression analysis of the selected genes is shown in Figure 3. The FD-treated groups (group 4 and 6) showed a significant decrease in the expression of *TP53*, *RAC1*, *COX-2*, *TWIST*, *CCND1*, and *EGFR* genes when compared with untreated group (group 2). Among all groups, a statistically significant difference (*p* < 0.05) was observed in all selected genes with the exception of *TP53* gene (Table 5).

## 4. Discussion

Animal models are regarded essential parts in investigating how disease progresses and how diagnostic or therapeutic protocols develop. The use of natural products such as green tea, fruit and vegetable extracts from grape, and apple and tomatoes for the chemoprevention and therapeutic of oral cancer has been gaining interest [5–7, 25]. To date, the antitumor activity of FD extract, a medicinal natural plant, on oral cancer has not been reported. In this study, an animal model that had been induced for oral cancer using 4NQO was used to determine the chemopreventive and chemotherapeutic activities of the FD extract.

It has been reported that when rats received 4NQO for 10 weeks at 20 ppm, the SCC incidence in the animal tongue was extremely reproducible (83%) at 26 weeks of 4NQO administration [26]. Depending on the dose and duration of the carcinogen being administered, 4NQO may trigger different types of dysplastic and neoplastic lesions with molecular and morphological alterations that may be related to the human carcinogenesis [27]. In the current study, 4NQO used at 20 ppm induced cancerous and precancerous morphological changes on the tongue epithelium after 22 weeks of cancer induction.

4NQO administration in animals for cancer induction can also result in substantial body weight loss due to the occurrence of oral cancer, as well as the lack of appetite, the inability to eat, and an increase in metabolic rate [28]. Similar to the previous reports, the use of 4NQO in this study produced a significant reduction of body weight in the cancer-induced animals. However, the weight reduction was controlled by the high dose of FD extract treatment.

To obtain further insight into the molecular mechanisms involved in the antitumor activity of FD extract in the 4NQO oral cancer animal model, we assessed the expression of selected genes and proteins associated with tumor growth activities such as cellular proliferation and cancer progression (*CCND1, EGFR*, and *COX-2*), cellular adhesion, and epithelial-mesenchymal transition (EMT) process (*β*–catenin, e-cadherin, *TWIST1*, and *RAC1*) and apoptosis (bcl2 and p53).

Cyclin D1 is a protein needed for the progression of the cell cycle G1 phase. Cyclin D1 overexpression promotes the transformation of a malignant phenotype that is associated with the progression of various types of cancer [29]. The cyclin D1 overexpression has been reported to be directly linked to the histopathological differentiation of OSCC [30].

This feature is also evident in this study where in the control group of rats induced for oral cancer, the cyclin D1 expression by immunohistochemistry was found in the parabasal and basal compartments of the keratinised epithelium. The expression of cyclin D1 was also found in the connective tissues, indicating invasion of the tumor cells into the underlying tissues. Inversely, administration of the FD extract at high dose in both the chemopreventive and chemotherapeutic groups decreased the cyclin D1 expression which was restricted to the basal compartment of the keratinised stratified squamous epithelium. This could indicate that the FD extract at high dose could have played a role in decreasing tumor aggressiveness. *CCND1* gene is a positive regulator of cell cycle [31], and the overexpression of *CCND1* in OSCC has been associated with a shorter survival rate of cancer patients [32, 33]. In addition, Wilkey et al. [34] reported in an animal study that *CCND1* overexpression increases the susceptibility of the mice towards oral carcinogenesis induced by 4NQO. Following induction of oral cancer in an animal 4NQO model, it was reported that the expression of *CCND1* decreased when the animals were exposed to substances with antitumor activity. Yoshida and his colleagues found that the administration of troglitazone (used to suppress the growth of tumors) had decreased the expression of *CCND1* in the 4NQO animal model [35].

Similarly, Naoi et al. [36] found that mikeside (shown to inhibit the proliferation of cancer cells) had significantly reduced the expression of *CCND1* and *COX-2* in the 4NQO animal model induced for oral cancer. In the present study, the effects of the FD extract on *CCND1* expression were investigated using IHC and RT^2^ profiler PCR array for both chemopreventive and chemotherapeutic studies. In line with the previous studies using substances with antitumor activity, *CCND1* expression was also found to have decreased in the FD extract treated groups following cancer induction using 4NQO. The results obtained were validated by both the IHC and RT^2^ profiler PCR array methods.

The upregulation of the *COX-2* gene has been linked to oral cancer and oral premalignant lesions. It was reported that cancer lesions at the later stage have a higher expression of the *COX-2* gene, as well as in patients with poor prognosis [37]. Ribeiro et al. [38] also reported that the *COX-2* expression is linked with the later stage of oral carcinogenesis, where the expression of *COX-2* has been found to be upregulated. In a related study, the mean expression of *COX-2* in OSCC was found to be 15.9 ± 6.7-fold greater than that in the adjacent tissues which consist of normal oral tissues [39]. A previous study assessing the antitumor effect of the apple extract reported that the *COX-2* expression had decreased in the apple extract-treated groups following cancer induction using 4NQO, in comparison with the control group of 4NQO alone [6]. Similarly, the *COX-2* expression by the RT^2^ profiler PCR array in this study was found to have decreased when treated with the FD extract when compared with the cancer control group for both types of experiment: chemopreventive and chemotherapeutic.

The epidermal growth factor receptor (*EGFR*) has been reported to be overexpressed in >80% of head and neck SCC [40]. The *EGFR* gene overexpression has also been correlated to several other types of cancer, including anal and lung cancers. The *EGFR* pathway when blocked has been reported to have an effect on the signalling pathways and can lead the cancer cells towards the apoptosis stage [41].

As pointed out by Singh et al. [42], the potential for these cancer cells to become invasive is eradicated through the inhibition of *EGFR*. Wali et al. [23] have shown that the painting of the rat's oral cavity with polyethylene glycol-8000 using a sable brush for up to 3–4 minutes has significantly lowered the expression of *EGFR* in the 4NQO rats. As for the present study, the results obtained are in line with those of the previous studies. It was found that there was a reduction in the expression of *EGFR* by the RT^2^ profiler PCR array in the FD extract-treated groups for both chemopreventive and chemotherapeutic experiments when compared with the 4NQO control group.

Ravi et al. [43] stated that the expression of the antiapoptotic bcl2 protein is closely correlated with the expression of p53. The overexpression of p53 has been reported in 55% and 75% of the North Indian patients with oral dysplasia and OSCC, respectively [44]. Scrobota et al. [45] discovered that the expressions of cyclin D1, ki-67, bcl2, p53,and p63 were found to have increased in line with the severity of the rats' dysplastic lesion when 4NQO was applied topically on the rats' tongues for 12 weeks. On the other hand, other studies have reported the lack of expression or a sporadic bcl2 expression in oral dysplasia [46, 47]. Ribeiro et al. [48] have shown that bcl2 has an important role in the initiation of SCC in the rats' tongues mucosa, but in well-differentiated SCC, induced by 4NQO for 20 weeks, the bcl2 expression was sometimes absent or reduced [48]. Other studies have also reported that bcl2 was overexpressed in poorly differentiated carcinomas [49, 50]. In the present study, the bcl2 expression between the FD extract groups and the cancer control group was observed to be not significant. It could be that the development of oral cancer in this study is associated with the well-differentiated type of OSCC, and hence this could have contributed to the nonsignificant expression of bcl2.

From a clinical perspective, patients with an increased expression of p53 may have a poor prognosis compared with cancer patients with a low expression of p53 [51]. Fong et al. [52] conducted a research investigating the effect of consuming zinc as a supplement for the oral carcinogenesis using 4NQO. They reported that zinc had decreased the expression of tumor markers such as cyclin D1, cox-2, and p53 when compared with the 4NQO control group. In this study, the FD extract treated groups for both the chemopreventive and chemotherapeutic experiments have shown an overexpression of p53 in the control cancer group (Group 2). However, the results were not significant for both the IHC and the RT^2^ profiler PCR molecular analysis methods. It has been reported that the *P*53 gene is mutated in about 50% of all types of human cancer, including oral cancer [53], and this could have contributed to the nonsignificant expression of p53 in this study.

The epithelial calcium-dependent adhesion molecules e-cadherin and *β*-catenin are cell adhesion proteins. A reduction in the cell adhesive proteins has been reported in tumor development [54] and in the progression of the head and neck SCC [55]. A decreased expression of *β*-catenin related to poor prognosis of breast cancer has also been reported [56]. El-Rouby [5] reported an increase in *β*-catenin and e-cadherin expressions when lycopene or tomato carotenoids were used to treat oral cancer in 4NQO-induced rats. Similarly, when the FD extract was used to treat oral cancer induced by 4NQO, it was observed that the expressions of the *β*-catenin and e-cadherin had increased.

Our study demonstrated that the FD extract had significantly reduced the *TWIST1* expression (*p* < 0.05) when compared with the control 4NQO group. *TWIST1* can act as a predictor of distant metastasis, plays an important role in the progression of OSCC during the EMT process, and is also involved in the later stage of cancer development [57]. Similar to our study, De Paiva Gonçalves et al. [58] reported that the *TWIST1* expression was found to have decreased, although nonsignificantly in the curcumin treatment group when compared with the control 4NQO rat group [58].

Rac1, a key protein that is involved in the transduction signalling pathway, belongs to the Ras superfamily of GTP-binding proteins [59]. The role of Rac1 in Ras transformation has been analysed by introducing constitutively active mutants [60–62]. It has been reported in lung cancer that *RAC1* overexpression is related to the EMT process with poor prognosis [63]. The overexpression of the *RAC1* gene in the breast cancer tissues has also been reported [64]. In this study, the *RAC1* gene was downregulated significantly (*p* < 0.05) in the FD extract treated group when compared with the control 4NQO group.

In conclusion, FD extract inhibited the proliferation of the cancer cells by decreasing the expression of *CCND1*, *EGFR*, and *COX-2*. Moreover, FD extract maintained epithelial polarity (cellular adhesion) and discouraged the aggressiveness toward OSCC by increasing the expression of e-cadherin and *β*-catenin and downregulate *TWIST1* and *RAC1* genes. It is concluded that the FD extract has the potential in being developed as a chemopreventive and chemotherapeutic agent for oral cancer therapy and future studies would be required to further elucidate the exact molecular pathway involved in the antitumor activity of FD extract in oral cancer.

## Figures and Tables

**Table 1:** Effect of FD on body weight of 4NQO-induced oral cancer rats.

Groups *N* = 7	Normal group (negative control)	Untreated 4NQO group (positive control)	Chemo preventive groups		Chemotherapeutic groups	
			FD 500	FD 250	FD 500	FD 250
Body weight (g)	^*∗*^551 ± 17	395 ± 00	^*∗*^479 ± 00	455 ± 00	440 ± 00	405 ± 00

^*∗*^The mean difference is significant at the 0.05 level compared with the 4NQO control group.

**Table 2:** Effect of FD on tongue tumor size in 4NQO-induced oral cancer in rats (a post hoc Dunnets test).

Group (I)	Tongue tumor volume (mm^3^) (mean ± SD)	*p* value
Group 2 (control)	87.03 ± 4.73	—
Group 3	60.91 ± 3.52	0.023^*∗*^
Group 4	40.69 ± 12.26	<0.001^*∗*^
Group 5	69.91 ± 1.80	0.170
Group 6	68.21 ± 32.46	0.114

^*∗*^ *p* value less than 0.05 (*p* < 0.05), significant value.

**Table 3:** Incidence of OSCC and precancerous lesions of rat's tongue treated with FD extract following the induction of oral cancer using 4NQO.

Group	No. of animals	Hyperplasia	Dysplasia	OSCC	*p* value
4NQO (group 2)	**7** (100%)	**0** (0.0%)	**1** (14.3%)	**6** (85.7%)	0.03^*∗*^
FD250 (group 3)	**7** (100%)	**1** (14.3%)	**3** (42.9%)	**3** (42.9%)	
FD500 (Group 4)	**7** (100%)	**4** (57.1 %)	**2** (28.6 %)	**1** (14.3%)	
FD250 (Group 5)	**7** (100%)	**0** (0.0%)	**5** (71.4%)	**2** (28.6%)	
FD500 (Group 6)	**7** (100%)	**0** (0.0%)	**6** (85.7%)	**1** (14.3%)	

^*∗*^ *p* value less than 0.05, (*p* < 0.05), significant value (chi-square test).

**Table 4:** Immunohistochemical evaluation of the FD effect on 4NQO-induced oral cancer in rats (a post hoc Tukey test).

IHC stain	Treated groups	Control group	*p* value
Cyclin D1	FD prevention	4NQO (control)	<0.001^*∗*^
	FD therapeutic		0.020^*∗*^
bcl2	FD prevention		0.622
	FD therapeutic		1.000
e-cadherin	FD prevention		<0.001^*∗*^
	FD therapeutic		0.040^*∗*^
*β*-catenin	FD prevention		<0.001^*∗*^
	FD therapeutic		0.017^*∗*^
p53	FD prevention		0.237
	FD therapeutic		0.149

^*∗*^ *p* value less than 0.05 (*p* < 0.05), significant value.

**Table 5:** Expression of genes in control (G2) and FD groups (G4 and G6) by RT^2^ PCR in rats administered with 4NQO.

	*CCND1*	*EGFR*	*COX2*	*RAC1*	*TP53*	*TWIST*
4NQO (G 2) (2^−ΔCt)	0.062	0.080	0.080	0.076	0.038	0.004
FD 500 (G 4) (2^−ΔCt)	0.022	0.009	0.008	0.020	0.011	0.001
Fold change	0.358	0.114	0.113	0.262	0.287	0.272
*p* value	0.036^*∗*^	0.037^*∗*^	0.038^*∗*^	0.016^*∗*^	0.067	0.039^*∗*^
FD 500 (G 6) (2^−ΔCt)	0.018	0.018	0.015	0.0188	0.011	0.002
Fold change	0.292	0.225	0.196	0.244	0.290	0.437
*p* value	0.028^*∗*^	0.049^*∗*^	0.046^*∗*^	0.015^*∗*^	0.068	0.063

^*∗*^ *p* value less than 0.05 (*p* < 0.05), significant value.

## Data Availability

The data that support the findings of this study are available from the corresponding author upon reasonable request.
